# Spin Seebeck mechanical force

**DOI:** 10.1038/s41467-019-10625-y

**Published:** 2019-06-13

**Authors:** Kazuya Harii, Yong-Jun Seo, Yasumasa Tsutsumi, Hiroyuki Chudo, Koichi Oyanagi, Mamoru Matsuo, Yuki Shiomi, Takahito Ono, Sadamichi Maekawa, Eiji Saitoh

**Affiliations:** 1 Advanced Science Research Center, Japan Atomic Energy Agency, Tokai, 319-1195 Japan; 2 Advanced Institute for Materials Research, Tohoku University, Sendai, 980-8577 Japan; 3 RIKEN Center for Emergent Matter Science, Wako, 351-0198 Japan; 4 Institute for Materials Research, Tohoku University, Sendai, 980-8577 Japan; 5 Kavli Institute for Theoretical Sciences, University of Chinese Academy of Sciences, Beijing, 100190 People’s Republic of China; 6 Quantum-Phase Electronics Center, The University of Tokyo, Tokyo, 113-8656 Japan; 7 Department of Applied Physics, The University of Tokyo, Tokyo, 113-8656 Japan; 8 Department of Mechanical and Aerospace Engineering, Tohoku University, Sendai, 980-8579 Japan

**Keywords:** Spintronics, Magnetic devices

## Abstract

Electric current has been used to send electricity to far distant places. On the other hand, spin current, a flow of electron spin, can in principle also send angular momentum to distant places. In a magnet, there is a universal spin carrier called a spin wave, a wave-type excitation of magnetization. Since spin waves exhibit a long propagation length, it should be able to send angular momentum that can generate torque and force at a distant place: a new function of magnets. Here we observe mechanical angular momentum transmission and force generation due to spin waves injected into Y_3_Fe_5_O_12_ by the spin-Seebeck effect. The spin-wave current, transmitted through a Y_3_Fe_5_O_12_ micro cantilever, was found to create a mechanical force on the cantilever as a non-local reaction of the spin-Seebeck effect. Spin-wave current can be generated remotely even in open circuits, and it can be used to drive micro mechanical devices.

## Abstract (web summary)

Spin current carrying angular momentum enables intriguing phenomena and many fascinating applications. Here the authors demonstrate that thermally driven spin Seebeck current can give rise to the mechanical torque when transmitting through a Y_3_Fe_5_O_12_ micro cantilever.

## Introduction

In 1915, Einstein and de Haas reported that an object starts rotating when it is magnetized: the Einstein-de Haas effect^1–3^. They explained the effect in terms of angular momentum conservation between magnetization and mechanical motion. A similar effect may arise from ferromagnetic resonance (FMR), where a magnet is rotated by a reaction of magnetization damping^4,5^. However, entire torque created by the Einstein-de Hass effect is limited by total spins in a sample, a situation which has made its application difficult. On the other hand, recently discovered spin-wave current^6–8^ can create a continuous flow of angular momentum^9^, which makes it possible to inject unlimited total angular momentum into a matter^10^.

The spin-Seebeck effect (SSE)^11–14^ is a practical way to generate such a powerful spin-wave current; when a part of a sample is heated, spin waves are created and flow out of the part. According to an analysis of SSE^15^, SSE can create much greater flux of angular momentum than the standard spin-pumping methods^16^, since SSE drives a broader energy range of spin waves^17,18^ than spin pumping.

**Fig. 1:**
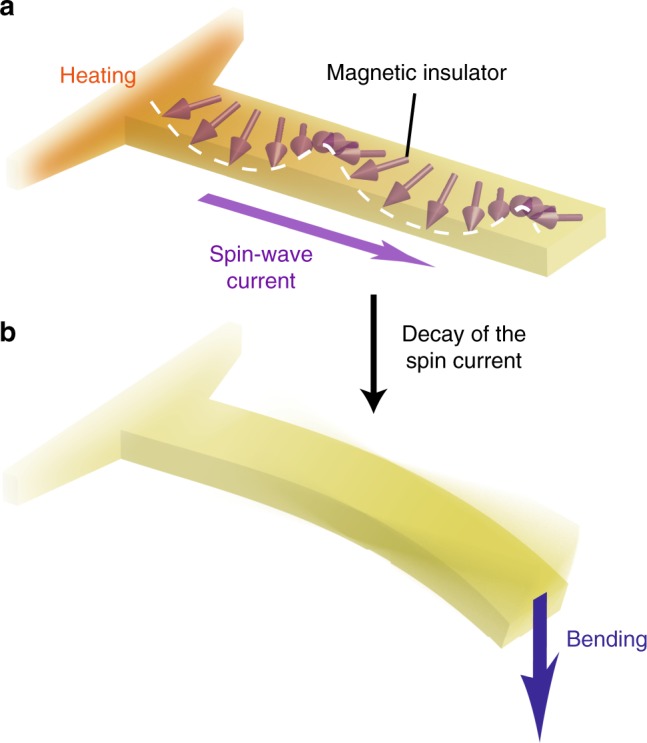
Schematic illustration of spin Seebeck mechanical force. **a** A schematic illustration of spin Seebeck effects. When a part of a magnetic insulator is heated, a spin wave is excited and flows out of the part: the spin-wave spin Seebeck effect. A spin wave carries angular momentum. **b** Relaxation of spin waves generates macroscopic mechanical torque and force due to the angular momentum transfer

Here we show that spin waves transmit angular momentum and create force to drive mechanical motion in an insulating magnet. In the present study, spin waves are injected into a small magnetic insulator Y_3_Fe_5_O_12_ (YIG). When the spin waves are relaxed in a part of the magnet, there should be its reaction on the magnet; due to the angular momentum conservation between spin waves and the lattice system of the magnet, the part of the magnet may start rotating (see Fig.1). Observing this unexplored spin-mechanical torque is our target in the present study.

## Results

### Sample description and characterization

**Fig. 2:**
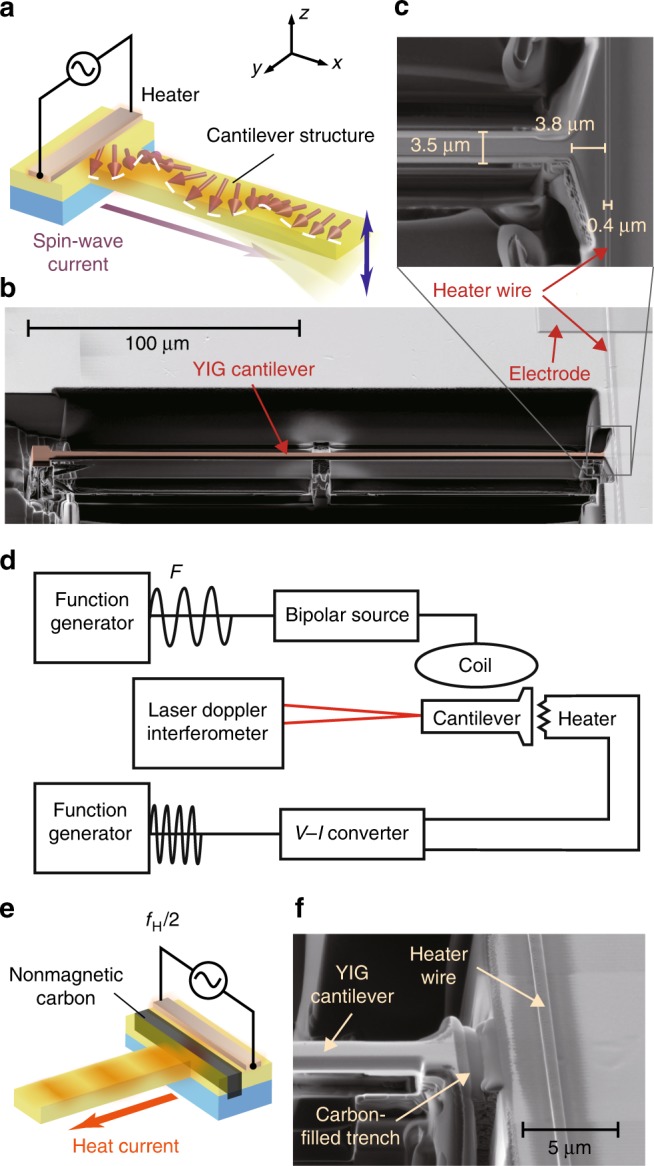
Sample system. **a** A schematic illustration of the sample used in the present study. The sample consists of a cantilever made of Y_3_Fe_5_O_12_(YIG) connected to an edge of a YIG film and a heater placed on the YIG film around the root of the cantilever. An electric current applied to the heater generates heat, which flows across the YIG film and the GGG substrate toward the sample holder. The heat current creates spin-wave (magnon) accumulation at the surface and the bottom of the YIG film. The accumulation injects spin current into the YIG cantilever connected around the surface of the film. **b** A scanning electron beam microscope (SEM) image of the YIG cantilever used in the present study. The heater wire is connected to a current source via the electrode pad. **c** A magnified view around the root of the cantilever. **d** A block diagram of the measurement system. Vertical fluctuation of the tip of the cantilever is measured by using a laser-Doppler interferometer. An a.c. heat with the frequency *f*_H_ is generated by applying an a.c. current with the frequency *f*_H_/2. An a.c. magnetic field with the frequency *F* is applied simultaneously. **e** A schematic illustration of the control sample, in which a trench filled with carbon was introduced between the heater and the cantilever (the black bar in the Figure). **f** A SEM image around the root of the control sample

Figure 2a, b shows a schematic illustration and a scanning electron microscope (SEM) image of an YIG cantilever used in the present study. The mechanical torque is observed as force acting on a micro-cantilever, one of the most sensitive force detectors^19–22^. When mechanical torque along the *y* axis acts on the cantilever, as shown in Fig. 2a, the cantilever bends along the *z* axis. The cantilever was fabricated by a focused-ion-beam (FIB) technique from a single-crystalline YIG with 3-µm-thick on a Gd_3_Ga_5_O_12_ (GGG) substrate^23^ (see “Methods” for details). The length, width, and thickness of the cantilever arm are 200, 3.5, and 1.6 μm, respectively. We put a thin heater wire on the bulk joint part made of YIG to which the cantilever is connected, as shown in Fig. 2c. By applying current pulses to the heater, the YIG joint is locally heated, creating spin waves. The spin waves then flow into the cantilever as a spin-wave current, a phenomenon called the spin-wave SSE, and it is expected to generate torque via the spin-wave relaxation in the cantilever. The distance between the wire and the cantilever, 3.8 μm, is shorter than the spin-diffusion length of spin waves in YIG, 8.7 μm^3^.

**Fig. 3:**
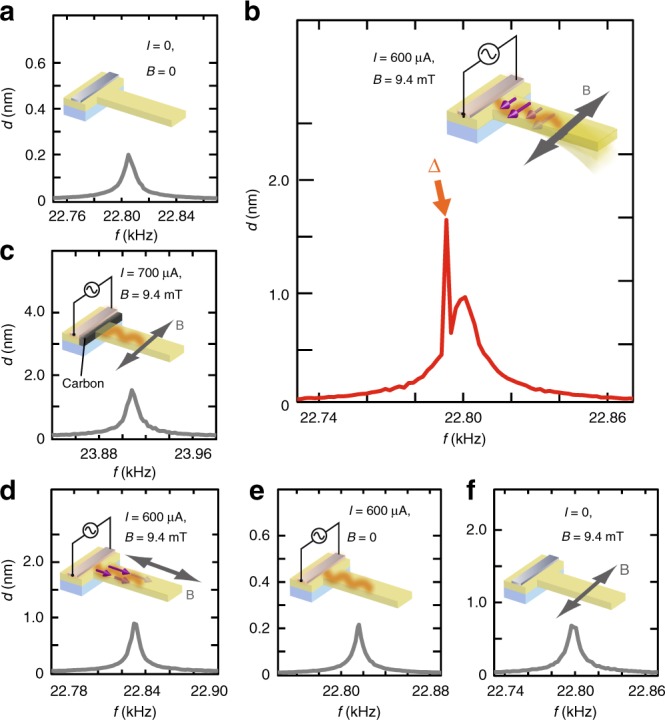
Amplitude of cantilever fluctuation *d* as a function of fluctuation frequency *f*. **a** Background thermal fluctuation. The spectrum was obtained without any external excitation. A broad peak around 22.8 kHz is the fundamental thermal vibration of the cantilever induced just by heat. **b** Fluctuation spectrum obtained with an a.c. current and an a.c. field perpendicular to the cantilever. The frequencies of the current *f*_H_/2 and the field *F* are 9.8960 kHz and 3.0010 kHz, respectively. A sharp peak is labeled as Δ. **c** Fluctuation spectrum obtained for the control sample with an a.c. current and an a.c. field perpendicular to the cantilever. The frequencies of the current *f*_H_/2 and the field *F* are 10.4272 and 3.0500 kHz, respectively. **d** Fluctuation spectrum obtained with an a.c. field parallel to the cantilever and an a.c. current. The frequency of the current *f*_H_/2 and the field *F* are 9.9038 and 3.0010 kHz, respectively. **e** Fluctuation spectrum obtained with an a.c. field perpendicular to the cantilever without current. The frequency of the field is 3.0010 kHz. **f** Fluctuation spectrum obtained with an a.c. current without fields. The frequency of the current is 9.8960 kHz

In Fig. 3a, we show the amplitude of cantilever fluctuation as a function of its frequency *f* measured without any external excitation to oscillation; the amplitude is simply due to thermal fluctuation. In the spectrum, a broad peak around *f*_0_ = 22.8 kHz appears. *f*_0_ is consistent with the numerically calculated frequency of the fundamental vibration of the cantilever (see Supplementary Note 1 for details). At the frequencies inside the broad peak, significant thermal fluctuation of the cantilever is always excited due to the room temperature heat, which means that, inside the peak, the cantilever is quite sensitive to external force due to the mechanical resonance. Taking advantage of the sensitivity, we performed dual-frequency a.c. measurements^22^ by applying a.c. spin-wave currents with the frequencies within the broad peak to detect spin-wave mechanical force (see “Methods” for details).

### Cantilever fluctuation measurements

We then applied heat pulses (a.c. heat) to the sample with the repetition frequency *f*_H_ = 19.792 kHz. Surprisingly, a clear sharp peak emerges at 22.793 kHz in the amplitude spectrum (shown as Δ in Fig. 3b) signaling unconventional cantilever motion superimposed on the broad thermal background peak. In the measurement, the external a.c. magnetic field is applied perpendicular to the cantilever with the modulation frequency *F* = 3.0010 kHz. The frequency at which the sharp peak Δ appears *f*_p_ = 22.793 kHz coincides with the frequency sum *f*_H_ + *F*, showing that the sharp peak Δ is due to cantilever motion synchronized with the heat-current pulses created in the cantilever. In all measurements, the d.c. component of the magnetic field is zero.

The sharp peak Δ has nothing to do with the conventional thermal expansion or distortion, but it is attributed to the spin-wave angular momentum transfer. We fabricated a sample in which a cantilever is magnetically isolated from the spin-wave injector but it is thermally coupled^24^, which we call the control sample. Figure 2e is a schematic illustration of the control sample: the root of the YIG cantilever is replaced with nonmagnetic carbon, which conducts heat well but blocks out spin waves. Because the thermal conductivity of carbon is comparable to that of YIG^25,26^, the control sample exhibits similar thermal properties to the YIG cantilever. Nevertheless, in the control sample, no sharp peaks are observed in the amplitude spectra even when the same heat pulses and fields are applied, while the broad peak corresponding to the background thermal vibration is almost unchanged as shown in Fig. 3c. The result indicates that the cantilever motion at the sharp peak is driven by the torque magnetically transmitted through the cantilever, ruling out phonon mediated effects as well as thermal stress. The similar behavior is observed in other samples, showing the universality of the present effect (see Supplementary Note 2 for details).

To double-check the spin-wave angular momentum transfer, we confirmed that the sharp peak Δ completely disappears when the magnetization of the YIG is directed parallel to the cantilever length by applying a field along the *x* direction, as shown in Fig. 3d. Since the angular momentum carried by the spin waves is directed along the magnetization, the fundamental vibration of the cantilever cannot be excited by the spin-wave angular momentum when the magnetization is along the cantilever length, which is consistent with the observed magnetization-direction dependence. We also confirmed that the sharp peak Δ disappears when either the external a.c. field or the heat pulses are switched off, as shown in Fig. 3e, f.

### Current and field dependence of cantilever fluctuation

**Fig. 4:**
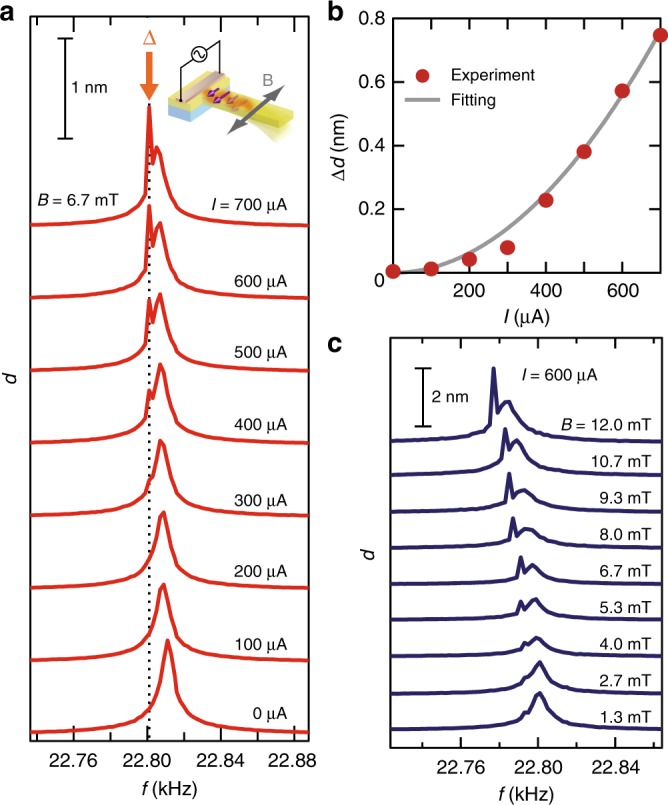
Current and field dependence of cantilever fluctuation *d*. **a** Amplitude of the cantilever fluctuation *d* at various values of the heating current *I*. The excitation frequency, 22.800 kHz, is shown by the dotted line in the figure. **b** Fluctuation amplitude of the cantilever at the excitation frequency as a function of the current amplitude *I*. The red disks represent the experimental data. The gray solid curve, which is proportional to *I*^2^, is a curve fitted to the data. **c** Amplitude of the cantilever fluctuation *d* at various values of the a.c. field strength. The current amplitude is 600 μA

The amplitude of the cantilever fluctuation *d* at various values of the heating current *I* is shown in Fig. 4a, where *f*_H_ *+* *F* is indicated as the dotted line. The intensity of the sharp peak Δ increases monotonically with increasing *I*. The amplitude calculated by subtracting background from *d* at the frequency *f*_H_ *+* *F*, *Δd*, is proportional to the heating power *I*^2^, as shown in the red disks and the solid curve in Fig. 4b. Since the spin-wave current intensity is proportional to the heating power *I*^2^, the experimental result is consistent with the torque generation due to the spin-wave SSE. Figure 4c shows the spectra of the cantilever oscillation at various external a.c. fields. The sharp peak was found to be suppressed at fields below 4.0 mT. In the field range, magnetic domains are introduced around the root of the cantilever, according to micro-magnetic simulation^27^ (see Supplementary Note 3 for details). Since a domain wall scatters spin waves^9^, the efficiency of the spin-wave transmission or the resultant torque should decrease in such a low field range, as shown in Fig. 4c. The change in the resonance frequency *f*_0_ with increasing field strength is attributed to the ΔE effect^28,29^, which refers to the modulation of Young’s modulus via magnetostriction, and we tuned the excitation frequency, *f*_H_ + *F*, to *f*_0_−10 Hz in the measurement.

## Discussion

The microscopic mechanism of the angular momentum transfer is likely composed of spin-orbit interaction, dipole-dipole interaction, and/or spin-rotation interaction^30–33^, but we phenomenologically assumed that the spin-wave angular momentum is totally transferred to the cantilever dynamics via the spin-wave relaxation, similar to the Einstein-de Haas effects and the mechanical rotation due to damping of FMR. By considering the mechanical boundary conditions, we estimated the force as |*F*_z_| ~ 10^−15^ N for the heating current *I* = 300 μA, which is consistent with the minimum detectable force by the cantilever at room temperature, *F*_min_ = 1.33 × 10^−15^ N (see Supplementary Note 4 for details). Thus, the longitudinal relaxation of the spin wave converts spin-wave angular momentum into mechanical force on the cantilever with *f*_p_ ~ 20 kHz^4,5^.

In magnets, spin-wave relaxation is sensitive to the magnetic shape anisotropy via the many-body scattering of magnons^28^, in our sample, the shape anisotropy in the thin cantilever arm is stronger than that in the bulk joint part. The magnons created in the joint part are thus strongly dissipated in the arm part, exerting torque on the cantilever. The shape anisotropy can be controlled in terms of shapes of a magnet, and one can design where a spin wave is created in a magnet and where it is converted to mechanical torque. Furthermore, the present mechanism can generate mechanical torque ceaselessly, different from the conventional Einstein-de Haas effects. These advantages can be applied to make various micro machines which can be driven from a distant place free from wiring, but using heat or microwaves.

## Methods

### Sample fabrication

The YIG cantilever used in the present study was fabricated with a dual beam FIB/SEM system (Versa3D DualBeam; FEI company) from a 3-µm-thick YIG (111) film epitaxially grown on a single-crystalline GGG substrate by a liquid-phase epitaxy method. First, we prepared the electrode pads by the electron-beam lithography. Next, we patterned a cantilever shape by focused Ga^+^ ion beam milling. To avoid magnetic-field disturbance from the YIG layer which surrounds the cantilever^21^, the surrounding YIG layer was removed about 30 µm away from the cantilever. Then, the Pt heater wire with the width of 0.4 µm was fabricated by the FIB deposition. The ends of the wire were connected to the electrode pads. Finally, the part under the cantilever was milled away by using an obliquely incident Ga^+^ ion beam. In the control sample, the trench at the root of the cantilever was shaped by Ga^+^ ion beam milling after the process explained above. The depth of the trench is greater than 5 µm, enough to cut the YIG layer. After the milling, the trench was filled with carbon by the FIB deposition.

### Measurement setup

In the present study, we measured vertical displacement of the cantilever by means of dual-frequency a.c. measurements based on a heterodyne detection method. The displacement was measured with a laser-Doppler interferometer (MSA-100-3D; Polytec, Inc.). During the measurements, an a.c. heat with the frequency *f*_H_ and an a.c. magnetic field with the frequency *F* were applied to exclude effects of thermal stress from the displacement. In the condition, the signal of the spin Seebeck mechanical force, which depends on both the a.c heat and the a.c field, appears at the frequency of *f*_H_ ± *F*, and any signals due to thermal stress, which are independent of the a.c. field, appear at the frequency of *f*_H_. Therefore, we can distinguish the spin Seebeck mechanical force from other artifacts originated in thermal effects. By tuning the excitation frequency *f*_H_ + *F* into the resonance band of the cantilever, the signal at *f*_H_ + *F* is resonantly amplified compared to off resonance bending. The signal at *f*_H_−*F* is out of range of the resonance band of the cantilever, and it is not detectable.

The a.c. heat was generated by applying an alternating current *I* with the frequency of *f*_H_/2 through the heater wire. The amplitude of *I* was controlled to be constant by using a *V-I* converter. The field strength was monitored with a Hall probe (HGT-2010; Lakeshore, Inc.). To separate the signal from a background, we tuned the excitation frequency *f*_H_ + *F* to be about 5 Hz less than the centre frequency of the cantilever fluctuation. Note that, in the single-frequency measurement in which an a.c. heat and a d.c. magnetic field were applied, the obtained signal does not depend on the field direction, which means that the signal of spin Seebeck mechanical force is completely hidden by thermal effects. All experiments were performed at room temperature in a high vacuum (10^−4^ Pa).

## Supplementary information

Supplementary Information

Peer Review

## Data Availability

The data that support the findings of this study are available from the corresponding author on request.
