# Supplementary material for: Spin Seebeck mechanical force
Source: Nat Commun. 2019 Jun 13;10:2616. doi: 10.1038/s41467-019-10625-y (PMC6565833; doi:10.1038/s41467-019-10625-y)
Supplement: Supplementary file 1 — Supplementary Information [file 41467_2019_10625_MOESM1_ESM.pdf]

Spin Seebeck mechanical force
Supplementary Information

K. Harii, Y-J. Seo, Y. Tsutsumi, H. Chudo, K. Oyanagi, M. Matsuo, Y. Shiomi, T. Ono, S. Maekawa, and E. Saitoh.

Supplementary Note 1: Characterization of cantilever

We numerically calculated the resonance frequency, f_0, of the cantilever by using the COMSOL multiphysics software.[1] The modeled cross-sectional shape of the cantilever is shown in Supplementary Figure 1. In the calculation, we used Young's modulus and the density values of $E = 1.9\times10^{11}$ Pa and $\rho = 5170$ kg/m^3, respectively.[2] The calculated resonance frequency of the first pendular mode is 25.3 kHz. Because the value is comparable to the experimental value of $f_0 = 22.8$ kHz in Fig. 3a, we assigned the sharp peak in Fig. 3a as the first-pendular mode.

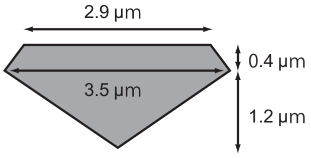

Supplementary Figure. 1: Cross section of a cantilever used in the numerical calculation.

Next, we estimated the minimum detectable force $\delta F_{\rm min}$, the equivalent force to thermal noise, defined as:[3]

$$\delta F_{\rm min} = \sqrt{\frac{4k_{\rm B}Tkw}{2\pi f_0 Q}}, \quad (1)$$

where $k_{\rm B}$ is the Boltzmann constant, T is the temperature, k is the spring constant, w is the measurement bandwidth, and Q is the quality factor. The spring constant of the cantilever $k = 1.74 \times 10^{-2}$ N/m was estimated from the resonance frequency:[4]

$$f_0 = \frac{(1.875)^2}{2\pi}\sqrt{\frac{k}{3L\rho A}}, \quad (2)$$

where L is the length of the cantilever, A is the cross-section area. The quality factor estimated from the resonance spectrum shown in Fig. 3a is $Q = 1140$. Using these values, we estimated the minimum detectable force of our cantilever at 300 K to be 1.33×10^{-15} N.

Supplementary Note 2: Spin Seebeck mechanical force for 300 μm cantilever

The amplitude of the cantilever fluctuation, d, as a function of its frequency, f, for a 300-μm cantilever without (2a) and with (2b) a carbon-filled trench is shown in Supplementary Figure 2. The direction of an a.c. magnetic field is perpendicular to the cantilever. In the figures, the excitation frequency, $f_H + F$, is indicated by a dotted line.

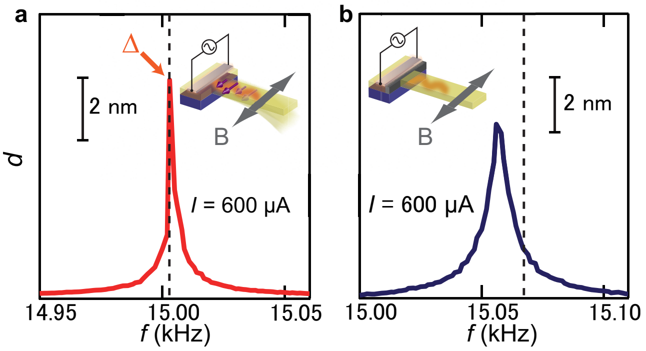

Supplementary Figure. 2: Amplitude of cantilever fluctuation for 300-μm cantilever with an a.c. heat and an a.c. field perpendicular to the cantilever. a, The amplitude obtained by using a cantilever witout a carbon-filled trench. b, The amplitude obtained by using a cantilever with a carbon-filled trench.

When the cantilever is directly connected to the YIG, a sharp peak labeled by Δ appears at the excitation frequency as shown in Supplementary Figure 2a. By contrast, as shown in Supplementary Figure 2b, no significant sharp peak appears at the excitation frequency when the cantilever is isolated by introducing a carbon-filled trench. The behavior is the same as that obtained for the 200-μm cantilevers described in the main text.

Supplementary Note 3: Micro-magnetic simulation of the cantilever

In this section, we show some results of the micromagnetic simulation to describe the magnetization distribution around the root of the cantilever.

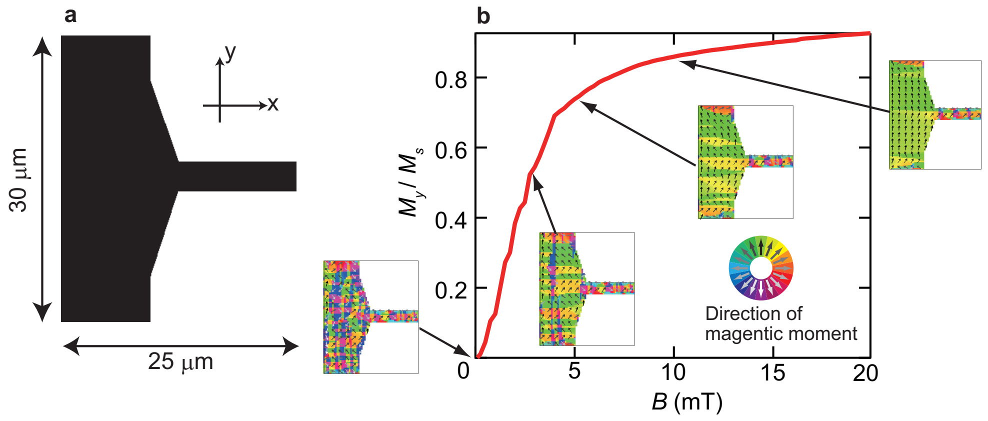

Supplementary Figure. 3: Result of micro-magnetic simulation. a, The shape and the size of the sample model. b, Field dependence of the y-component of the total magnetization divided by the saturation magnetization. (insets) Magnetic domain structures at various fields. The hue of the painted area represents the direction of magnetic moment.

The result of a micro magnetic simulation conducted using the OOMMF software[5] is shown in Supplementary Figure 3. We calculated magnetic moments in the 2D area shown in Supplementary Figure S3a to discuss magnetic domain structures around the root of the cantilever. The right half of the area represents the cantilever arm, and the left half the root of a cantilever. In the simulation, an external magnetic field, B, was applied in the y-direction shown in Supplementary Figure 3a. Supplementary Figure 3b shows the field dependence of the y-component of the total magnetization, M_y, divided by the saturation magnetization, M_s. M_y/M_s increases steeply from $B = 0$ to $B = 5$ mT, and is almost saturated above 5 mT. The behavior corresponds to the domain annihilation around the root area as shown in the insets to Supplementary Figure S3b. The result implies that the transmission of spin waves is suppressed below 5 mT, which is in good agreement with the experimental results.

Supplementary Note 4: Creation of mechanical torque by spin waves

In this section, we derive mechanical torque to drive a $Y_3Fe_5O_{12}$ (YIG) cantilever motion as a reaction of spin-Seebeck effect. When spin waves injected by the spin-Seebeck effect diffuse in the cantilever, the spin angular momentum is transferred into the cantilever and the mechanical torque is induced due to the total angular momentum conservation.

1. Spin waves by spin-Seebeck effect

Firstly, we describe spin waves in YIG injected by the a.c. spin-Seebeck effect from a Pt heater. The spin-wave, magnon, transport theory is based on the Boltzmann equation without external forces in the relaxation time approximation:

$$\boldsymbol{v_k} \cdot \boldsymbol{\nabla} n_k(\boldsymbol{r}) = -\frac{n_k(\boldsymbol{r}) - n_k^0}{\tau_k}, \tag{3}$$

where the magnon number in thermal equilibrium n_k^0 is given by the Bose-Einstein distribution $n_k^0 = [\exp(\hbar\omega_k/k_{\mathrm{B}}T) - 1]^{-1}$ for the wave vector $\boldsymbol{k}$, energy $\hbar\omega_k$, and group velocity $\boldsymbol{v}_k = \partial\omega_k/\partial\boldsymbol{k}$. Here, τ_k is the k-magnon relaxation time. The distribution of the magnon number $n_k(\boldsymbol{r})$ can be considered in the steady state because the excitation frequency $f_{\mathrm{H}} + F$ is of the order of kHz in our measurement.

The magnon accumulation is defined as the density of magnons in excess of thermal equilibrium:

$$\delta n^{\mathrm{m}}(\boldsymbol{r}) = \int \frac{d\boldsymbol{k}}{(2\pi)^3} [n_k(\boldsymbol{r}) - n_k^0], \tag{4}$$

and the spin-wave current density is written as[6–8]

$$\boldsymbol{j}^{\mathrm{m}}(\boldsymbol{r}) = \hbar \int \frac{d\boldsymbol{k}}{(2\pi)^3} \boldsymbol{v}_k [n_k(\boldsymbol{r}) - n_k^0]. \tag{5}$$

Since the spatial distribution of the magnon accumulation $\delta n_k(\boldsymbol{r}) \equiv n_k(\boldsymbol{r}) - n_k^0$ obeys the Boltzmann equation (Supplementary Equation 3), the spin-wave current consists of two contributions, $\boldsymbol{j}^{\mathrm{m}} = \boldsymbol{j}^{\nabla T} + \boldsymbol{j}^{\delta n}$, where

$$\boldsymbol{j}^{\nabla T}(\boldsymbol{r}) = -\hbar \int \frac{d\boldsymbol{k}}{(2\pi)^3} \tau_k \frac{\partial n_k^0}{\partial T} \boldsymbol{v}_k [\boldsymbol{v}_k \cdot \boldsymbol{\nabla} T] \tag{6}$$

originates from the temperature gradient and

$$\boldsymbol{j}^{\delta n}(\boldsymbol{r}) = -\hbar \int \frac{d\boldsymbol{k}}{(2\pi)^3} \tau_k \boldsymbol{v}_k [\boldsymbol{v}_k \cdot \boldsymbol{\nabla} \delta n_k(\boldsymbol{r})] \tag{7}$$

rises in the magnon accumulation.

When the x-direction is taken to the direction of the temperature gradient, $j_x^{\nabla T}$ is given by $j_x^{\nabla T} = -C\partial T/\partial x$ with

$$C \equiv \hbar \int \frac{d\boldsymbol{k}}{(2\pi)^3} \tau_k v_{kx}^2 \frac{\partial n_k^0}{\partial T}. \tag{8}$$

Note that the spin-wave current owing to the temperature gradient is insignificant in our experiment because a YIG cantilever magnetically isolated from a spin-wave injector, a Pt heater, cannot be driven only by temperature difference. This consequence is consistent with recent reports that the spin-wave current rising in the magnon accumulation is dominant on a micron scale and at room temperature. [9–11]

Then, we focus on the spin-wave current sourced in the magnon accumulation. According to the spirit of linear response theory, the deviation of the magnon distribution $\delta n_k(\boldsymbol{r})$ in Supplementary Equation 7 is expanded in terms of the magnon energy $\epsilon_k = \hbar\omega_k$. In order to avoid the divergence of the magnon accumulation δn^{m} in Supplementary Equation 4 at $\epsilon_k = 0$, we expand

$$n_k(\boldsymbol{r}) = n_k^0 + n_k^0 \epsilon_k g(\boldsymbol{r}) \tag{9}$$

up to the first order of ϵ_k,[12] where $g(\boldsymbol{r})$ is a spatial distribution to be determined by the solution of the boundary value problem. By using Supplementary Equation 9 in Supplementary Equation 4, one can show that

$$\delta n^{\mathrm{m}}(\boldsymbol{r}) = I_0 g(\boldsymbol{r}), \tag{10}$$

where the integral

$$I_0 \equiv \int \frac{d\boldsymbol{k}}{(2\pi)^3} n_k^0 \epsilon_k \tag{11}$$

does not diverge even for a gapless magnon dispersion. Since $\delta n_k(\boldsymbol{r}) = (n_k^0 \epsilon_k / I_0)\delta n^{\mathrm{m}}(\boldsymbol{r})$, Supplementary Equation 7 gives a magnon diffusion current

$$j_x^{\delta n}(x) = -\hbar D_{\mathrm{m}} \frac{\partial \delta n^{\mathrm{m}}(x)}{\partial x} \tag{12}$$

with the magnon diffusion coefficient

$$D_{\mathrm{m}} \equiv \frac{1}{I_0} \int \frac{d\boldsymbol{k}}{(2\pi)^3} \tau_k v_{kx}^2 n_k^0 \epsilon_k, \tag{13}$$

where we consider the one-dimensional magnon distribution along the x-axis. Besides, the magnon accumulation obeys a diffusion equation,[6–8]

$$\frac{\partial^2 \delta n^{\mathrm{m}}(x)}{\partial x^2} = \frac{\delta n^{\mathrm{m}}(x)}{l_{\mathrm{m}}^2} \tag{14}$$

with the magnon diffusion length l_{m}. Finally, the magnon diffusion current density is given by

$$j_x^{\delta n}(x) = \hbar \frac{D_{\mathrm{m}}}{l_{\mathrm{m}}} \delta n^{\mathrm{m}}(0) e^{-x/l_{\mathrm{m}}}, \tag{15}$$

where $\delta n^{\mathrm{m}}(0)$ is the magnon accumulation at a contact with the Pt heater, namely, the source of the spin-Seebeck effect.

2. Mechanical torque due to diffusion of spin waves

From the spin-wave diffusion current in Supplementary Equation 15, we can derive mechanical torque due to the total angular momentum conservation. The continuity equation for the spin density $s(\boldsymbol{r})$,

$$\frac{ds(\boldsymbol{r})}{dt} = -\boldsymbol{\nabla}\cdot\boldsymbol{j}^{\delta n}(\boldsymbol{r}), \tag{16}$$

gives relaxation of the spin angular momentum density owing to diffusion of spin waves obeying Supplementary Equation 15. The total angular momentum conservation,

$$\frac{d\boldsymbol{L}}{dt} = -\int d\boldsymbol{r}\frac{d\boldsymbol{s}(\boldsymbol{r})}{dt}, \tag{17}$$

leads to mechanical torque $\boldsymbol{T} = d\boldsymbol{L}/dt$.

We consider a YIG cantilever used in the present experiment as a one-dimensional system along the x-axis in $0 < x < L$ with $L = 200$ μm. The polarization axis of magnons by the external a.c. magnetic field is taken to be the y-axis. The total angular momentum conservation in Supplementary Equation 17 gives force on the cantilever per unit length, f_z, as

$$\int_0^L dx x f_z(x) = -A\int_0^L dx\frac{\partial j_x^{\delta n}(x)}{\partial x} \approx A j_x^{\delta n}(0), \tag{18}$$

because $L \gg l_{\rm m}$ with $l_{\rm m} \approx 8.7$ μm at room temperature,[13] where $A = 3.38$ $\mu{\rm m}^2$ is the cross section of the cantilever. Since the spin angular momentum is transferred into the mechanical torque near the root of the cantilever within the magnon diffusion length $l_{\rm m}$, we assume that f_z is applied to an element of the cantilever at $x = l_{\rm m}/2$. Then, the shearing force acting on the cantilever is obtained by

$$|F_z| = 2\frac{A}{l_{\rm m}} j_x^{\delta n}(0). \tag{19}$$

Based on the experimental results in Refs. 9,14 and a numerical simulation in Ref. 15, the magnon diffusion current density at a contact with the Pt heater can be estimated at $j_x^{\delta n}(0) \sim 10^{-10}$ J/m^2 with a charge current $I = 100$ μA on the Pt heater. Therefore, in this condition, we estimate the force acting on the cantilever at $|F_z| \sim 10^{-16}$ N. This force should be proportional to heating power I^2 by the charge current on the Pt heater.

Supplementary references

[1] https://www.comsol.com/structural-mechanics-module/
[2] H. M. Chou, and E. D. Case, Mater. Sci. Eng. **100**, 7 (1988).
[3] Y. J. Seo, M. Toda, and T. Ono, J. Micromech. Microeng. **25**, 045015 (2015).
[4] L. D. Landau and E. M. Lifshitz, "Theroy of Elasticity 3rd edition" p. 117.
[5] http://math.nist.gov/oommf/
[6] S. S.-L. Zhang and S. Zhang, Phys. Rev. Lett. **109**, 096603 (2012).
[7] S. S.-L. Zhang and S. Zhang, Phys. Rev. B **86**, 214424 (2012).
[8] S. M. Rezende, R. L. Rodríguez-Suárez, R. O. Cunha, A. R. Rodrigues, F. L. A. Machado, G. A. Fonseca Guerra, J. C. Lopez Ortiz, and A. Azevedo, Phys. Rev. B **89**, 014416 (2014).
[9] J. Shan, L. J. Cornelissen, N. Vlietstra, J. Ben Youssef, T. Kuschel, R. A. Duine, and B. J. van Wees, Phys. Rev. B **94**, 174437 (2016).
[10] J. Shan, L. J. Cornelissen, J. Liu, J. Ben Youssef, L. Liang, and B. J. van Wees, Phys. Rev. B **96**, 184427 (2017).
[11] B. L. Giles, Z. Yang, J. S. Jamison, J. M. Gomez-Perez, S. Vélez, L. E. Hueso, F. Casanova, and R. C. Myers, Phys. Rev. B **96**, 180412(R) (2017).
[12] S. Rezende, R. Rodríguez-Suárez, R. Cunha, J. L. Ortiz, and A. Azevedo, J. Magn. Magn. Mater. **400**, 171 (2016).
[13] We used the magnon diffusion length estimated from the experiments in Ref. 14, in which the spin-wave damping modulation[16–18] affected by the spin Seebeck effect is renormalized into the diffusion length.
[14] L. J. Cornelissen, J. Liu, R. A. Duine, J. Ben Youssef, and B. J. van Wees, Nat. Phys. **11**, 1022 (2015).
[15] L. J. Cornelissen, K. J. H. Peters, G. E. W. Bauer, R. A. Duine, and B. J. van Wees, Phys. Rev. B **94**, 014412 (2016).
[16] Y. Tserkovnyak, S. A. Bender, R. A. Duine, and B. Flebus, Phys. Rev. B **93**, 100402(R) (2016).
[17] L. Lu, Y. Sun, M. Jantz, and M. Wu, Phys. Rev. Lett. **108**, 257202 (2012).
[18] C. Safranski, I. Barsukov, H. K. Lee, T. Schneider, A. A. Jara, A. Smith, H. Chang, K. Lenz, J. Linder, Y. Tserkovnyak, M. Wu, and I. N. Krivorotov, Nat. Commun. **8**, 117 (2017).
