# Supplementary material for: Spin Seebeck mechanical force
Source: Nat Commun. 2019 Jun 13;10:2616. doi: 10.1038/s41467-019-10625-y (PMC6565833; doi:10.1038/s41467-019-10625-y)
Supplement: Supplementary file 2 — Peer Review [file 41467_2019_10625_MOESM2_ESM.pdf]

Reviewers' comments:

Reviewer #1 (Remarks to the Author):

This is an interesting paper demonstrating that spin current carried by thermal magnons can transfer angular momentum to the lattice and thereby induce rotation of the lattice a distance away from the spin current source. The result is new and significant, and the experimental evidence is convincing. In my opinion, this paper should be published in the Nature Communications. I have several questions and suggestions to clarify/improve the presentation in the manuscript:

- The need for a dual-frequency detection is not explained in sufficient detail. The authors state that this technique employing magnetic field modulation excludes the mechanical displacement effects due to thermal stress. However, the explanation of why and how this exclusion takes place is lacking. A much more detailed explanation, perhaps in the supplemental material, is needed.

- The direction of the applied dc magnetic field in this experiment does not appear to be explicitly stated. Is it always collinear with the ac modulation magnetic field or is it always parallel to the y-axis, or applied in some other direction?

- The images in Fig. 1 are of low resolution – this makes it difficult to see various parts of the device in the SEM images.

- Why is the cantilever so much longer than the spin wave decay length? Would not the effect be amplified in a shorter cantilever?

- What is the origin of the ac field-induced resonance shift in Fig. 4c?

- Spin Seebeck current injected into the cantilever can modify the effective damping of spin waves of the system (Y. Tserkovnyak et al. Phys. Rev. B 93, 100402(R), L. Lu et al., Phys. Rev. Lett. 108, 257202 (2012), C. Safranski et al., Nat. Commun. 8, 117 (2017)), which can change backflow of angular momentum from the cantilever into the unpatterned YIG film. Are these effects of any importance for the system studied in this paper?

- There is a typo in Fig. 2 should be “bipolar” not “bipoler”

- The suggested similarity of the long-range spin signal propagation to electric signal propagation in the abstract is an overstatement. Spin current effects are still important in microscopic devices only, including the present study. I suggest to revise the abstract accordingly.

Reviewer #2 (Remarks to the Author):

This paper reports the experimental observation of mechanical bending of a $Y_3Fe_5O_{12}$ (YIG) cantilever due to the relaxation of spin waves or magnons. The main idea is that the relaxation of the spin waves leads to a transfer of angular momentum from the spin system to the lattice of the YIG cantilever, and this momentum transfer then results in a mechanical rotation or bending of the cantilever. The spin waves were injected by the spin-Seebeck effect (SSE). The bending of the YIG cantilever was measured by a laser doppler interferometer. The experiments were supported by micro-magnetic simulations and theoretical analyses.

I find this work interesting, but I also have a concern about the novelty of this work. There are already a number of papers reporting about magnon-relaxation produced mechanical rotation in magnetic thin

films, including the following two:
J. Appl. Phys., Vol. 89, No. 11, 1 June 2001
Appl. Phys. Lett., Vol. 78, No. 16, 16 April 2001
In my opinion, the physics in these two papers is pretty much the same as in this manuscript, although the experimental configurations are not the same. In their case, uniform magnons in a Permalloy thin film cantilever (not a YIG cantilever) pass angular momentum to the lattice of the Permalloy cantilever and thereby cause the Permalloy cantilever to bend up or down. The bending was measured by a laser technique, as in this work. Note that those two papers are not cited in this manuscript, and I suggest the authors to cite them.

I would think a flip in the direction of the external magnetic field should result in an opposite mechanical rotation or bending. Can the authors demonstrate this? Since the ac magnetic fields were used during the experiments, the authors might already have some data on this.

The authors wrote that the thickness of the cantilever is 1.6 micron, but it is not clear whether this is the thickness of the YIG film or the entire thickness of the YIG/GGG bilayer.
It is not clear whether the magnons are produced only by SSE in the Pt wire? Does the heating in the structure excites thermal magnons in the YIG cantilever?

What is "delta E" in line 118?

In the first paragraph, the authors compared the entire torque in the Einstein-de Hass effect and the possible torque due to spin waves and stated that spin waves can "inject unlimited total angular momentum into a matter." This is true, but what really matters is the rate of spin injection. In the second paragraph, the authors stated that "SSE can create much greater flux of angular momentum than the standard spin-pumping methods, since SSE drives a broader frequency range of spin waves." Yes, the spin waves driven by SSE have a broader frequency range, but how about the coherency and amplitude of the SSE-driven spin waves? Some explanations on these statements should be useful for non-expert readers.

Reviewer #3 (Remarks to the Author):

The authors have demonstrated the Einstein-de Hass effect by exploiting the Spin Seebeck effect. The authors fabricated a cantilever from an insulating ferromagnet. By generating heat at the root, they argue that a spin wave current will flow towards the cantilever. Under this condition, they find that a sharp peak emerges within the broad thermal resonance spectrum of the cantilever. This is interpreted as the signature of the spin wave converting its angular momentum to the mechanical rotation of the cantilever.

The experiments are solid and well thought out. The authors demonstrate:
1) The peak disappears when the heat current is blocked by a carbon layer
2) The peak also disappears when a magnetic field is applied parallel to the beam
3) The peak amplitude scales with I^2, showing the relation to heating.

In my view, the paper deserves publication, despite the following:

All the experiments done can be considered necessary but not 'sufficient' to establish the claim of a spin current converting its angular momentum into a mechanical motion. My biggest concern comes from the fact that the spin dynamics under the applied magnetic field should be in the GHz range. The authors have also mentioned it. This should mean that the conversion of angular momentum at a few kHz should be extremely inefficient if not non-existent and should also be damped completely by the cantilever's own vibration spectrum. The authors need to provide a detailed discussion of why this is

not the case. The authors should also consider doing supporting experiments such as BLS, perhaps on a larger structure, to probe the spin waves themselves. Also, why is there no peak at fH-F?

Authors' response to Reviewer #1

This is an interesting paper demonstrating that spin current carried by thermal magnons can transfer angular momentum to the lattice and thereby induce rotation of the lattice a distance away from the spin current source. The result is new and significant, and the experimental evidence is convincing. In my opinion, this paper should be published in the Nature Communications. I have several questions and suggestions to clarify/improve the presentation in the manuscript:

(Reviewer #1's Question/Comment 1)

- *The need for a dual-frequency detection is not explained in sufficient detail. The authors state that this technique employing magnetic field modulation excludes the mechanical displacement effects due to thermal stress. However, the explanation of why and how this exclusion takes place is lacking. A much more detailed explanation, perhaps in the supplemental material, is needed.*

(Authors' Answer 1)

Following the comment, we added an introduction to the dual-frequency method in the revised Methods section and detailed information about the thermal effects (page 5, line 28-35).

(Reviewer #1's Question/Comment 2)

- *The direction of the applied dc magnetic field in this experiment does not appear to be explicitly stated. Is it always collinear with the ac modulation magnetic field or is it always parallel to the y-axis, or applied in some other direction?*

(Authors' Answer 2)

We thank the reviewer for the valuable comments. Firstly, we would like to show experimental results in which a d.c. magnetic field and an a.c. spin current are applied. In the measurement, we used a short cantilever with the resonance frequency ~100 kHz.

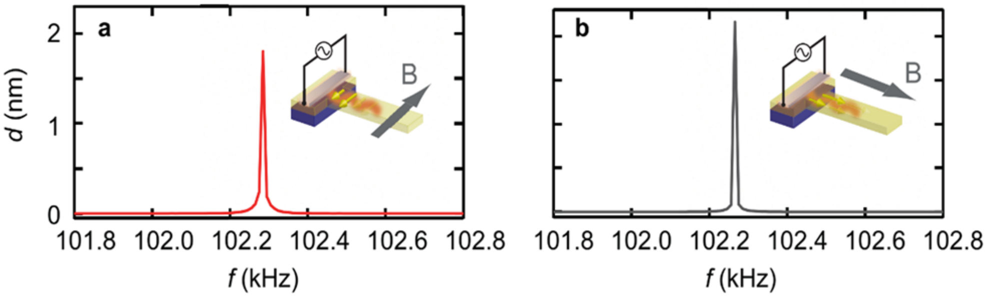

Figure R1

By applying a.c. heat whose frequency is the same as the cantilever's resonance frequency, a peak appears at the frequency of the a.c. heat. The peak intensity is almost identical between when the field direction is perpendicular (a) and parallel (b) to the cantilever, which means that the origin of the peak has nothing to do with cantilever's magnetization. We thus concluded that the spin Seebeck mechanical force is completely hidden by the thermal effects in the d.c. field measurement.

Based on the results, we decided to apply an a.c magnetic field instead of the d.c. field where the dual-frequency method is exploited. We made an electromagnet with a ferrite yoke which can generate a strong a.c. magnetic field enough to change the direction of YIG's magnetization. By using the dual-frequency method, the frequency of the spin Seebeck mechanical force, which depends on both heat and magnetic fields, is different from the frequency of the thermal effects, which depend only on heat. Therefore, we can obtain the signal of the spin Seebeck mechanical force separated from the thermal effects.

In response to the reviewer's comment, we explained this point in the revised text and the Methods section (page 3, line 11, page 6, line 5-9).

(Reviewer #1's Question/Comment 3)

- *The images in Fig. 1 are of low resolution – this makes it difficult to see various parts of the device in the SEM images.*

(Authors' Answer 3)

In response to the reviewer's comment, we improved the resolution of the figures.

(Reviewer #1's Question/Comment 4)

- *Why is the cantilever so much longer than the spin wave decay length? Would not the effect be*

amplified in a shorter cantilever?

(Authors' Answer 4)

Thank you very much for the comment. In order to enhance the displacement of the cantilever induced by spin angular momentum, we used the much longer cantilever than the spin wave decay length. According to Eq. (S1), minimum detectable force of a cantilever is proportional to $k^{1/2}$, where k is a spring constant, which means that a cantilever with smaller spring constant has higher sensitivity. A cantilever's spring constant strongly depends on the length of the cantilever. For example, k of a cantilever with rectangular cross section can be written as $k = (E\ t^3\ w)/(4\ l^3)$, where E is the Young's modulus. t, w, and l are the thickness, width, length of the cantilever, respectively. That means a long cantilever has small k. We thus employed a long cantilever to increase the sensitivity.

(Reviewer #1's Question/Comment 5)

- *What is the origin of the ac field-induced resonance shift in Fig. 4c?*

(Authors' Answer 5)

The resonance shift in Fig. 4c can be explained by the change in Young's modulus due to magnetostriction: the delta-E effect. For example, when magnetization is not parallel to an applied field, Young's modulus decreases due to the delta-E effect, and the resonance frequency decreases. In the revised manuscript, we improved the explanation on the effect and added some references to the main text (page 4, line 15-16, page7, line 30-33).

(Reviewer #1's Question/Comment 6)

- *Spin Seebeck current injected into the cantilever can modify the effective damping of spin waves of the system (Y. Tserkovnyak et al. Phys. Rev. B 93, 100402(R), L. Lu et al., Phys. Rev. Lett. 108, 257202 (2012), C. Safranski et al., Nat. Commun. 8, 117 (2017)), which can change backflow of angular momentum from the cantilever into the unpatterned YIG film. Are these effects of any importance for the system studied in this paper?*

(Authors' Answer 6)

We used the experimentally obtained value of the magnon diffusion length shown in Ref. 3, in which the modification of the effective damping is automatically included. It is known that the spin-current induced modulation of the damping is very small, and it is irrelevant to the observed phenomena. According to the reviewer's comment, we have mentioned the modification of the effective damping of magnons in a footnote of Supplementary Information D (Supplementary Information, page 4, line 36-37, line

40-43).

(Reviewer #1's Question/Comment 7)

- *There is a typo in Fig. 2 should be "bipolar" not "bipoler"*

(Authors' Answer 7)

Thank you very much for the comment. We corrected the typo.

(Reviewer #1's Question/Comment 8)

- *The suggested similarity of the long-range spin signal propagation to electric signal propagation in the abstract is an overstatement. Spin current effects are still important in microscopic devices only, including the present study. I suggest to revise the abstract accordingly.*

(Authors' Answer 8)

Thank you very much for the constructive suggestion. Following the comment, we improved the sentences in the abstract to provide more proper information for non-expert readers.

Authors' response to Reviewer #2

This paper reports the experimental observation of mechanical bending of a Y3Fe5O12 (YIG) cantilever due to the relaxation of spin waves or magnons. The main idea is that the relaxation of the spin waves leads to a transfer of angular momentum from the spin system to the lattice of the YIG cantilever, and this momentum transfer then results in a mechanical rotation or bending of the cantilever. The spin waves were injected by the spin-Seebeck effect (SSE). The bending of the YIG cantilever was measured by a laser doppler interferometer. The experiments were supported by micro-magnetic simulations and theoretical analyses.

(Reviewer #2's Question/Comment 1)

I find this work interesting, but I also have a concern about the novelty of this work. There are already a number of papers reporting about magnon-relaxation produced mechanical rotation in magnetic thin films, including the following two:

J. Appl. Phys., Vol. 89, No. 11, 1 June 2001

Appl. Phys. Lett., Vol. 78, No. 16, 16 April 2001

In my opinion, the physics in these two papers is pretty much the same as in this manuscript, although the experimental configurations are not the same. In their case, uniform magnons in a Permalloy thin film cantilever (not a YIG cantilever) pass angular momentum to the lattice of the Permalloy cantilever and thereby cause the Permalloy cantilever to bend up or down. The bending was measured by a laser technique, as in this work. Note that those two papers are not cited in this manuscript, and I suggest the authors to cite them.

(Authors' Answer 1)

We would like to thank the reviewer for the comment and suggestion on the references. We added the two papers to our references and added some comments on them (page 4, line 27-19, page 8, line 4-7). The novelty of our study is the first observation of torque *transport* by spin-wave currents. The important point we demonstrated is that a spin wave generates mechanical motion at a place spatially separated from the place where it is created. To our knowledge, such mechanical torque transport has never been reported.

(Reviewer #2's Question/Comment 2)

I would think a flip in the direction of the external magnetic field should result in an opposite mechanical rotation or bending. Can the authors demonstrate this? Since the ac magnetic fields were used during the experiments, the authors might already have some data on this.

(Authors' Answer 2)

Thank you very much for pointing this out. We tried to measure the static bending induced by a d.c. magnetic field and a d.c. spin current, but the non-resonant signal is too small to be detected. In the a.c. measurements, it was practically impossible to determine the sign of the bending direction since phase locking is too difficult in the dual-frequency method. We mentioned this point in the revised version (page 5, line 33-35, page 5, line 5-9).

(Reviewer #2's Question/Comment 3)

The authors wrote that the thickness of the cantilever is 1.6 micron, but it is not clear whether this is the thickness of the YIG film or the entire thickness of the YIG/GGG bilayer.

(Authors' Answer 3)

In response to the reviewer's comment, we mentioned the thickness of the YIG not only in the Methods section but also in the revised main text (page 2, line 16). The thickness of the YIG film is 3 μm, and the cantilever is made up of YIG alone.

(Reviewer #2's Question/Comment 4)

It is not clear whether the magnons are produced only by SSE in the Pt wire? Does the heating in the structure excites thermal magnons in the YIG cantilever?

(Authors' Answer 4)

As the reviewer pointed out, we also had a question about the origin of the magnons that generate cantilever motion. Motivated by this question, we performed the carbon-filled-cantilever experiment (Fig. 3c). The vibration signal disappears in the carbon-filled sample, and we concluded that the magnon current in our experiment is mainly generated near the Pt wire and it is diffused from the beneath of the wire to the cantilever.

(Reviewer #2's Question/Comment 5)

What is "delta E" in line 118?

(Authors' Answer 5)

The delta-E effect means change of Young's modulus due to magnetostriction. To clarify it, we added a sentence on the delta-E effect and some references to the main text (page 4, line 15-16, page 7, line 30-33).

(Reviewer #2's Question/Comment 6)

In the first paragraph, the authors compared the entire torque in the Einstein-de Hass effect and the possible torque due to spin waves and stated that spin waves can "inject unlimited total angular

momentum into a matter." This is true, but what really matters is the rate of spin injection.

(Authors' Answer 6)

We agree that the maximum rate of the spin injection is an important issue. In this study, as described in the Supplementary Information D, we assumed that the magnon current density (rate of angular-moment injection) is ~10^{-10} J/m^2 at the heater current *I* = 100 μA. Based on the assumption, the maximum rate of spin injection in our study is estimated to be ~5 ×10^{-9} J/m^2. But we cannot apply *I* > 700 μA since we have to prevent the sample from breaking; we did not check the exact upper limit of the spin injection rate in our sample. Improvement of the upper limit of the spin-injection rate is an important future task.

(Reviewer #2's Question/Comment 7)

In the second paragraph, the authors stated that "SSE can create much greater flux of angular momentum than the standard spin-pumping methods, since SSE drives a broader frequency range of spin waves." Yes, the spin waves driven by SSE have a broader frequency range, but how about the coherency and amplitude of the SSE-driven spin waves? Some explanations on these statements should be useful for non-expert readers.

(Authors' Answer 7)

Thank you very much for the comment. In response to the reviewer's comment, we improved the explanation to avoid misunderstanding. In the spin Seebeck effect, spin waves are thermally excited. Therefore, the excited spin waves are incoherent with distributed frequencies. We changed "frequency range" to "energy range" in the revised main text (page 1, line 33).

Authors' response to Reviewer #3

The authors have demonstrated the Einstein-de Hass effect by exploiting the Spin Seebeck effect. The authors fabricated a cantilever from an insulating ferromagnet. By generating heat at the root, they argue that a spin wave current will flow towards the cantilever. Under this condition, they find that a sharp peak emerges within the broad thermal resonance spectrum of the cantilever. This is interpreted as the signature of the spin wave converting its angular momentum to the mechanical rotation of the cantilever.

The experiments are solid and well thought out. The authors demonstrate:

1) *The peak disappears when the heat current is blocked by a carbon layer*
2) *The peak also disappears when a magnetic field is applied parallel to the beam*
3) *The peak amplitude scales with I^2, showing the relation to heating.*

In my view, the paper deserves publication, despite the following:

(Reviewer #3's Question/Comment 1)

All the experiments done can be considered necessary but not 'sufficient' to establish the claim of a spin current converting its angular momentum into a mechanical motion. My biggest concern comes from the fact that the spin dynamics under the applied magnetic field should be in the GHz range. The authors have also mentioned it. This should mean that the conversion of angular momentum at a few kHz should be extremely inefficient if not non-existent and should also be damped completely by the cantilever's own vibration spectrum. The authors need to provide a detailed discussion of why this is not the case. The authors should also consider doing supporting experiments such as BLS, perhaps on a larger structure, to probe the spin waves themselves.

(Authors' Answer 1)

We thank the reviewer for the comment. The angular-momentum transfer from spins to lattice system is caused by the "longitudinal" relaxation of magnetization precession, since the lattice dynamics is much slower. When the precession is excited steadily, the strength of the longitudinal relaxation keeps constant, in other words, a "dc angular-momentum transfer" appears. On the other hand, GHz precession represents the transverse component of the spin relaxation. In our study, the amplitude of the thermal spin waves in the cantilever was modulated with kHz frequency. It gives rise to kHz-modulation of the dc "longitudinal" angular-momentum transfer (not GHz). We performed some measurements with different frequencies, and confirmed that the peak position changes in response to the frequency change. Following the reviewer's

comment, we added some explanations to the revised main text and references on this point (page 4, line 24, 27-29).

(Reviewer #3's Question/Comment 2)

Also, why is there no peak at fH-F?

(Authors' Answer 2)

Thank you very much for the comment. In our experiment, f_H-F is out of range of the resonance band (shown in Fig. 3a) of the cantilever, and it is not detectable. In response to the reviewer's comment, we added a sentence on this point to the Methods section (page 5, line 34-35).

REVIEWERS' COMMENTS:

Reviewer #1 (Remarks to the Author):

In my opinion, the authors have addressed the concerns raised by the referees. I recommend this manuscript for publication.

Reviewer #2 (Remarks to the Author):

My main concern was about the similarity between this work and the JAP 2001 and APL 2001 papers I mentioned. I can see that the authors have cited those two papers on page 4, but somehow they did not provide any comments/discussions about those previous works, possibly due to the length limit. I would suggest the authors add some comments so that the novelty of this work is very well justified for the general readers. Except that, I think this is a pretty nice work.

Reviewer #3 (Remarks to the Author):

The authors have answered my questions satisfactorily. I recommend the paper for publication.

(Authors' response to Reviewer #2)

My main concern was about the similarity between this work and the JAP 2001 and APL 2001 papers I mentioned. I can see that the authors have cited those two papers on page 4, but somehow they did not provide any comments/discussions about those previous works, possibly due to the length limit. I would suggest the authors add some comments so that the novelty of this work is very well justified for the general readers. Except that, I think this is a pretty nice work.

(Authors' Answer)

Thank you very much for esteeming the quality of the experimental work and for recommending publishing our manuscript.

Following the suggestion, we added the sentence about the JAP 2001 and APL 2001 papers in the main text.

(Authors' response to Editor)

Thank you very much for your editorial effort. Following your comments, we shortened the abstract into 150 words. We also corrected the affiliation, the references, and the end note section in the main text. In Supplementary Information, we changed the format of the figure titles, the style of equations' number, the text style of the subsection and added the section title "Supplementary references".
